# Tribological performance of organic molybdenum in the presence of organic friction modifier

**DOI:** 10.1371/journal.pone.0252203

**Published:** 2021-06-10

**Authors:** Weiwei Wang, Zhuangzhuang Liu, Qimin Song, Xindi Zhang, Shengkai Jiao, Yao Xu, Quanda Xu, Dezun Sheng

**Affiliations:** Ocean School, Yantai University, Yantai, China; University of Vigo, SPAIN

## Abstract

The tribological performance of organic molybdenum in the present of organic friction modifier was investigated in this study. Three types of organic friction modifiers were selected, which are Glycerol monooleate, Pentaerythritol and *N*,*N*-Dimethylhexadecylamine. The organic molybdenum are MoDTC, MoDDP and molybdenum amide. Friction coefficient and wear were studied in block-on-ring test rig with steel test specimens. Experimental results indicate the Pentaerythritol shows synergistic effect with MoDTC in wide range temperature, while increased the friction coefficient of molybdenum amide in high temperature. *N*,*N*-Dimethylhexadecylamine shows synergistic effect with molybdenum amide, while hindered the friction reduction performance of MoDTC in low temperature. The presence of Glycerol monooleate reduced friction coefficient of MoDTC in low temperature, while increased the friction coefficient of molybdenum amide in most situations. All the tested organic friction modifiers improved the friction reduction performance of MoDDP. Most of the tested organic friction modifiers reduced the wear of organic molybdenum. The PT shows the best anti-wear performance with MoDTC. The tribo-chemical products in test specimens lubricated with different lubricant formulas indicate that the presences of Pentaerythritol promotes the production of MoS_2_ in MoDTC. *N*,*N*-Dimethylhexadecylamine promotes the production of MoS_2_ in molybdenum amide. The side products of MoO_1.6_S_1.6_ and Cr/MoS_2_ of MoDDP in high temperature lead to high friction coefficient.

## Introduction

Organic molybdenum has long been applied as an effective additive for reducing friction [1–4], which shows significant friction reduction performance in steel, iron and other surface [5, 6]. Therefore, it draws extensive attention for the study on friction reduction mechanism.

Typical organic molybdenum are Molybdenum dithiocarbamates (MoDTC), molybdenum dialkyl dithiophosphate (MoDTP), molybdenum dialkyldithiophosphate (MoDDP) and molybdenum amide. In general, the tribo-chemical decomposition of organic molybdenum and the generation of molybdenum disulphide (MoS_2_) are the key to reducing friction coefficient. By means of Raman spectroscopy, Khaemba et al analyzed the role played by MoDTC decomposition in steel/steel contacts [7]. According to the analytical results, the products of MoDTC decomposition include MoS_2_, FeMoO_4_ and sulfur-rich molybdenum compounds, as well as MoSx (x>2). FeMoO_4_ is derived from the side reaction between iron oxides and molybdenum compounds at low temperatures and low MoDTC concentrations. As for the tribo-chemical product, surface roughness will have an impact on the tribo-chemical production of MoDTC. A rough surface contributes to the increased generation of MoS_2_ from MoDTC, while a smooth surface leads to the mixture of MoS_2_, MoSx (x>2) and FeMoO_4_, which indicates the partial decomposition of MoDTC [8].

Lubricants are the mixture of base oil and different functional additives. The organic molybdenum will be impacted by the presence of other additives. Both synergies and antagonisms can be found between functionalized additive and organic molybdenum [6].

Syngernistic tribological effect could be produced when MoDTC is mixed with zinc dialkyldithiophosphate (ZDDP). The research results indicate that the lubricant formulations and tribo-chemical reaction films could make a difference to the deformation of subsurface layers, which plays an important role in the anti-wear performance [9]. Syngernistic tribological effect could also be found when mixing sulfur- and phosphorus- free molybdenum amide (MA) with ZDDP [10]. As indicated by the results of four-ball machine, the 2%wt MA and 1%~1.25% ZDDP are optimal for friction reduction and anti-wear performance. The similar synergistic results were also found by Cai T and Zhang J [11, 12]. The mixture of 0.5% MA and 0.5% ZDDP reduced the friction coefficient to almost 35% compared with pure MA and ZDDP, with amorphous MoS_3_ and MoS_2_ as the intermediate product of MA and ZDDP mixture. In severe friction condition, the main product is MoS_2_ [13]. In addition, fatty triamine was also identified as effective in enhancing the performance in friction reduction [14]. The MoS_2_ long sheets were found on friction surface, which aligned along the sliding direction [15]. Yan et al compared the tribological behaviors of sulfur and phosphorus free organic molybdenum, ZDDP and MoDTC. When the concentration of sulfur- and phosphorus- free organic molybdenum reached 2% wt, the friction coefficient and wear scar were minimized. The wear scar of four-ball test with organic molybdenum was the lowest compared with ZDDP and MoDTC [16]. Compared with MoDTC, the friction coefficient was reduced by 25% in the presence of highly sulfurised moly-trimer additive and MA additive [17]. The production of MoS_2_/MoS_2−*x*_O_*x*_ shows solid lubricant character on steel surfaces. The MoS_2_/MoS_2−*x*_O_*x*_ sheets are mainly embedded in an oxygen-rich amorphous matrix while the tribo-chemical product of MoDTC-ZDDP mixture is polyphosphate/phosphate glass matrix.

The mixture of GMO, MoDTC, and ZDDP shows syngergistic friction reduction performance in PAO base oil and palm trimethylolpropane ester, which reduced the friction coefficient by 30%~50% [18, 19].

Apart from syngersitic effect, some additives may have adverse effect on the performance of organic molybdenum in reducing friction, especially organic friction modifiers, which have a polar functional group to form adsorption boundary film on friction surfaces and reduce friction coefficient. Typically, organic friction modifiers include ester, amide, fatty acid and long chain fatty amine. The polar group-containing organic friction modifiers can improve the solubility of polar additives in non-polar base oil [20]. It is demonstrated that the increasing ester concentrations will increase the friction coefficient of MoDTC in steel/iron contacts [21]. Though the friction coefficient of lubricant containing ester and MoDTP can lead to low friction coefficient at the beginning of friction test, the low friction phenomenon is not long-lasting in the presence of ester [22]. Therefore, in order to optimize the composition in lubricant, it is necessary to figure out the influence of organic friction modifier to organic molybdenum compound.

In this paper, an investigation was conducted about the tribological performance of organic molybdenum in the presence of organic friction modifier. The sulfur- and phosphorus- free molybdenum amide (MA), the sulfur-containing Molybdenum dithiocarbamates (MoDTC), and the sulfur—phosphorus containing molybdenum dialkyldithiophosphate (MoDDP) were selected as the organic molybdenum. The Glycerol monooleate (GM), Pentaerythritol (PT) and N,N-Dimethylhexadecylamine (AM) were selected as organic friction modifiers. Sliding friction was performed on block-on-ring test ring. The friction coefficient and wear of test specimens were recorded. The interaction mechanism among organic friction modifiers and organic molybdenum were discussed.

## Materials and methods

### Lubricants

**Fig 1:**
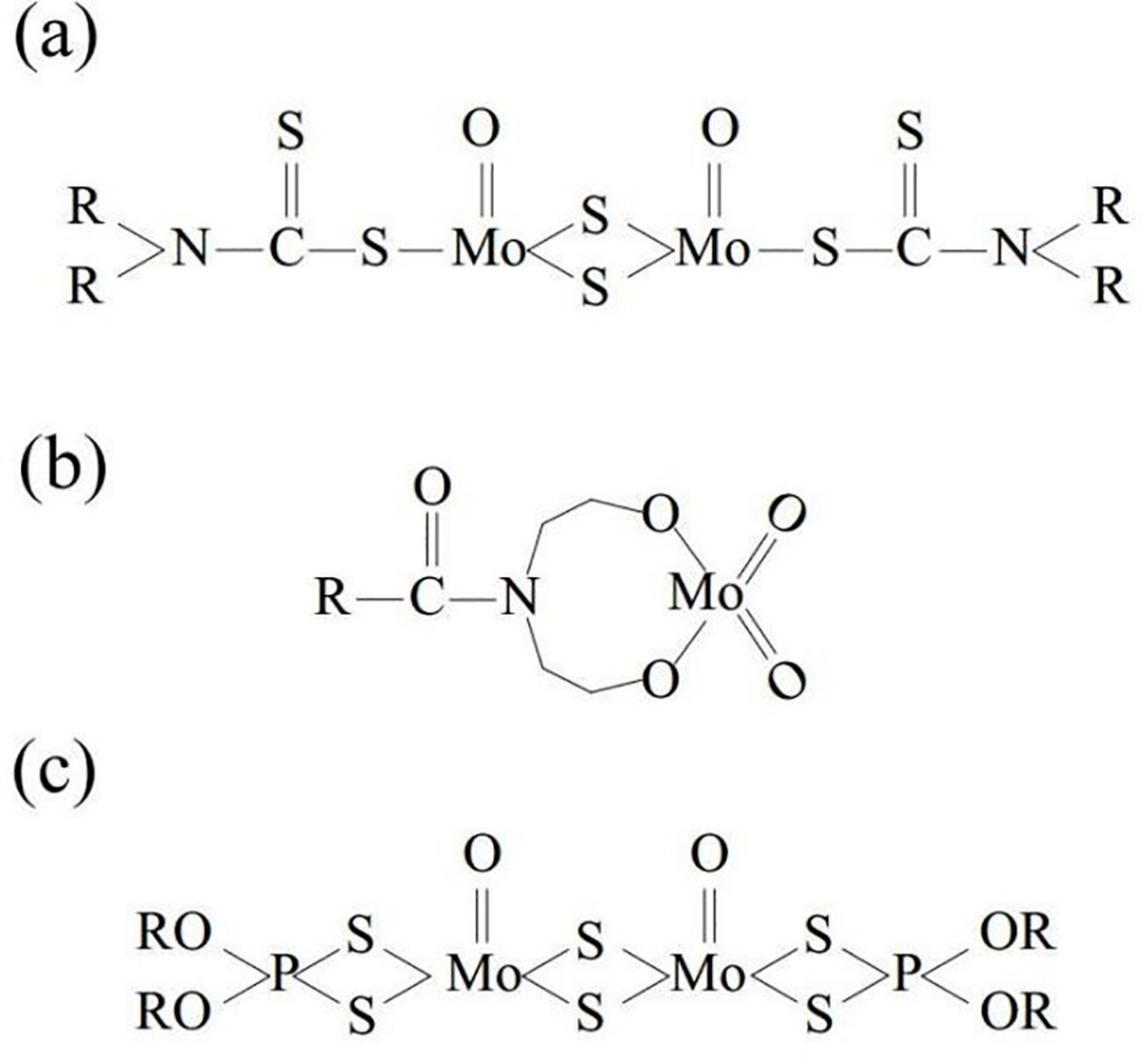
Molecular structures of organic molybdenum given by the suppliers. (a) molybdenum dithiocarbamate (MoDTC), (b) molybdenum amide (MA), and (c) Molybdenum dialkyldithiophosphate (MoDDP).

The organic molybdenum friction modifiers used for friction study includes molybdenum dithiocarbamate (MoDTC), molybdenum dialkyldithiophosphate (MoDDP) and molybdenum amide (MA), which were provided by Minglanchem. Co. Ltd. The molecular structures of organic molybdenums given by the suppliers are shown in Fig 1.

The organic friction modifiers include Glycerol monooleate (purity 97.7%), Pentaerythritol (purity 95%) and N,N-Dimethylhexadecylamine (purity 98%). The organic friction modifiers, which contain no sulfur and phosphorus, were supplied by Shanghai Aladdin Bio-Chem Technology Co. Ltd. Group I mineral base oil was selected for its wide application in machinery lubrication. Because Group I base oil was distilled from crude oil, impurities as sulfur often exist in the base oil. The sulfur concentration of base oil in this study was 0.11%wt, which was analysed by OSA 4 metallab on-site oil analyzer.

1 wt% organic molybdenum and 1 wt% organic friction modifier were added into the base oil, which is an effective concentration [23]. The base oil and additives were weighed by analytical balance, then mixed thoroughly with Magnetic Stirrer. Viscosity of test oil samples were measured with capillary viscometer. For the description to be simplified in the following sections, the abbreviations of base oil and additives are listed in Table 1. The test oil formulations are shown in Table 2.

**Table 1:** Base oil and additive abbreviations.

Abbreviation	Full name	Category
BO	Base oil	Group I mineral oil
MC	MoDTC	Organic molybdenum
MP	MoDDP	Organic molybdenum
MA	Molybdenum amide	Organic molybdenum
GM	Glycerol monooleate	Organic friction modifier
PT	Pentaerythritol	Organic friction modifier
AM	*N*,*N*-Dimethylhexadecylamine	Organic friction modifier

**Table 2:** Test oil formulations.

Abbreviation	Organic friction modifier, wt%	Organic molybdenum, wt%	Base oil, wt%	Viscosity, 40°C mm^2^·s	Viscosity, 100°C mm^2^·s	Viscosity Index
BO	0.0	0.0	100.0	34.1	5.8	112
BO-MC	0.0	MoDTC, 1.0	99.0	34.9	6.0	118
BO-MC-GM	1.0	MoDTC, 1.0	98.0	36.2	6.4	126
BO-MC-PT	1.0	MoDTC, 1.0	98.0	34.6	6.1	122
BO-MC-AM	1.0	MoDTC, 1.0	98.0	34.1	6.0	121
BO-MA	0.0	MA, 1.0	99.0	34.3	6.0	119
BO-MA-GM	1.0	MA, 1.0	98.0	35.1	6.3	128
BO-MA-PT	1.0	MA, 1.0	98.0	34.8	6.2	127
BO-MA-AM	1.0	MA, 1.0	98.0	34.2	6.0	122
BO-MP	0.0	MoDTP, 1.0	99.0	35.5	6.0	114
BO-MP-GM	1.0	MoDTP, 1.0	98.0	36.8	6.3	120
BO-MP-PT	1.0	MoDTP, 1.0	98.0	35.8	6.2	121
BO-MP-AM	1.0	MoDTP, 1.0	98.0	35.2	6.1	118

### Test specimens

**Fig 2:**
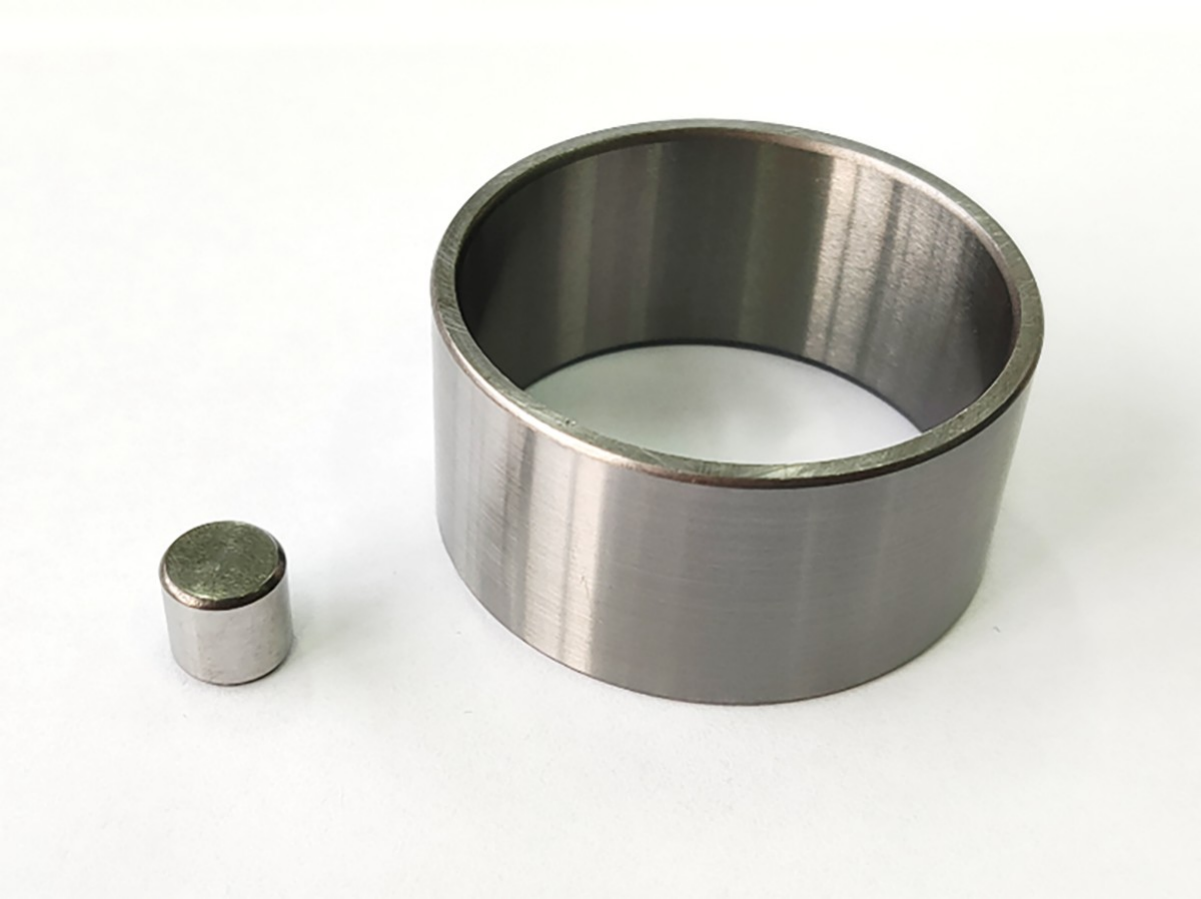
The ring and block of test specimens.

The ring and block of test specimens were of GCr15 steel, which is typically used for fabricating bearings and friction specimens in tribological test. The ring and block are shown in Fig 2. The test specimens have a hardness of HV 751 for the ring and block. The surface roughness Ra of ring and block are 0.1 μm and 0.3 μm. The ring is 45mm in inner diameter, 50mm in outer diameter and 25mm in height. The block is a cylinder which is 10 mm in height and diameter.

### Experimental apparatus

**Fig 3:**
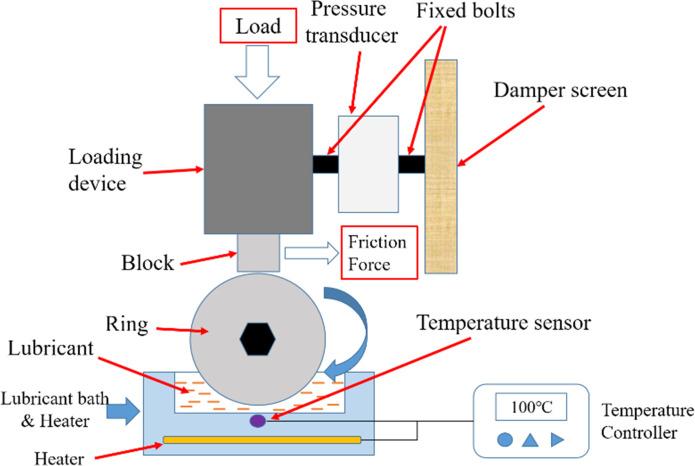
Schematic of test rig.

**Fig 4:**
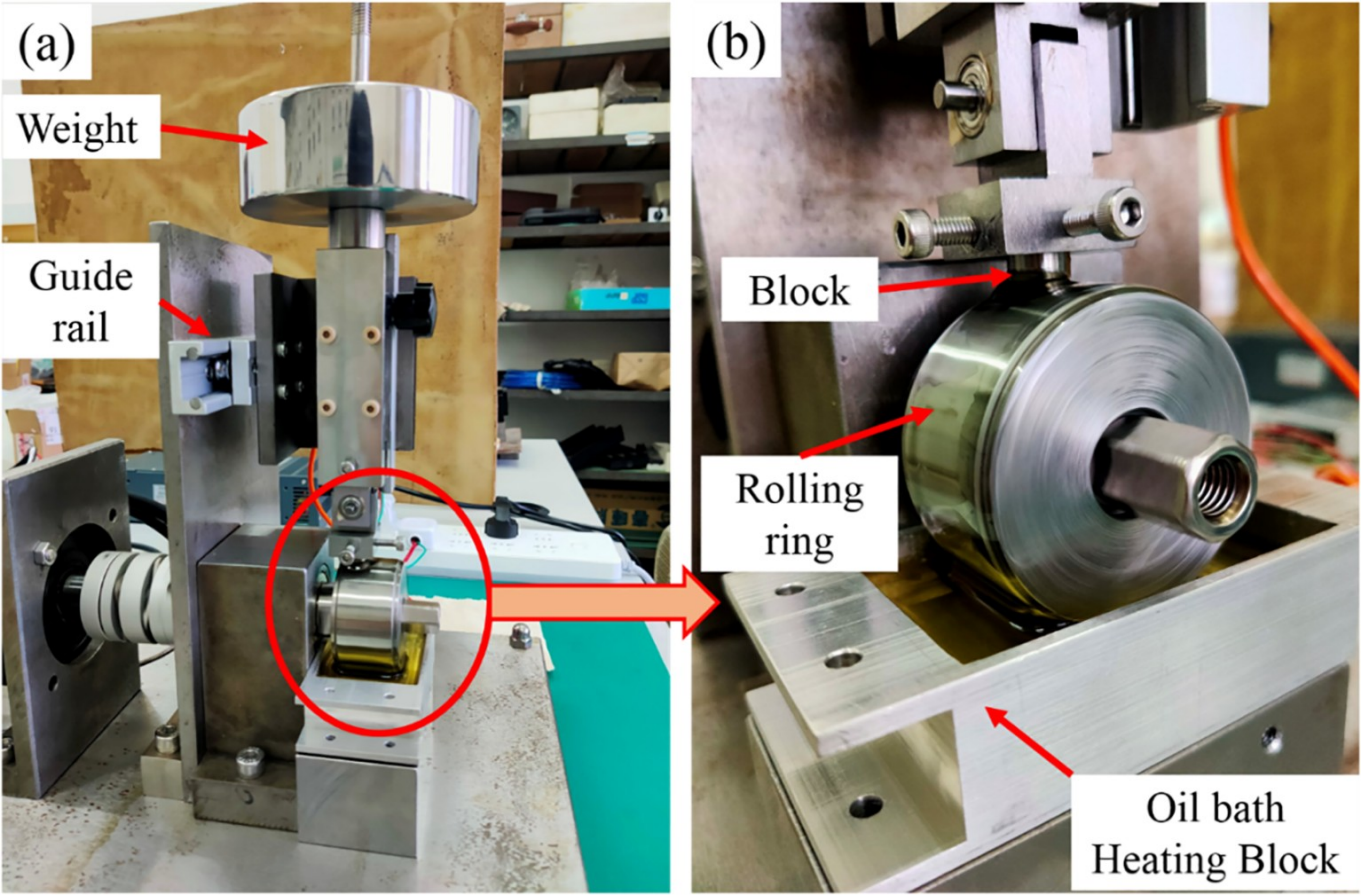
Photos of test rig.

Block-on-ring test rig was fabricated to verify the lubricating performance. Block-on-ring is a typical line contact, which represents the contact state of cylinder liner and piston ring in diesel and gasoline engine. Therefore, it has been widely used in lubrication performance test. The schematic of test rig is illustrated in Fig 3. Photos of test rig is shown in Fig 4. The ring was fitted on the rolling shaft driven by the servo motor, while the block was fixed on the loading device. A normal load was applied on the top of block. Sliding friction appeared between ring and block when shaft rolling. The loading device was fixed on a guide rail, which can move freely along the friction force direction. When the friction force pushing the loading device, the friction force was detected by the pressure transducer fixed between the loading device and damper screen. Lubricant bath was located beneath the ring. With the rolling of ring, the lubricant contained in the lubricant bath was entrained onto the friction surface.

Heater and temperature sensor were embedded in the lubricant bath, temperature of lubricant bath was controlled by the temperature controller. The oil entrained in the contact surface is hard to be detected, therefore, the temperature of lubricant bath represents the lubricant temperature in this paper. The temperature can be controlled from 40°C to 150°C, which is typical temperature for engine bearing in cold starting and long term running. The speed at which the shaft spun can be controlled from 0 r/min to 3000 r/min. The load was applied with weights, which are varied from 10 to 100N. The pressure transducer Longlv-LLLBM, which measures the friction force, is produced by Shanghai Longlv Electronic Technology Co., Ltd. The measuring range is ±100N, with accuracy of ±0.1N. Coupled with the charge amplifier, the friction force signal was read by data Acquisition Card YAV-USB 8AD Plus, which is produced by Wuhan YAV Electronic Technology Co., Ltd. Friction data was measured by every second, totally 21600 data points.

The friction coefficient was obtained with Eq 1

μ=F/N (1)

Where *μ* is friction coefficient, *F* is friction force (N), *N* is applied load (N)

### Experimental procedure

**Fig 5:**
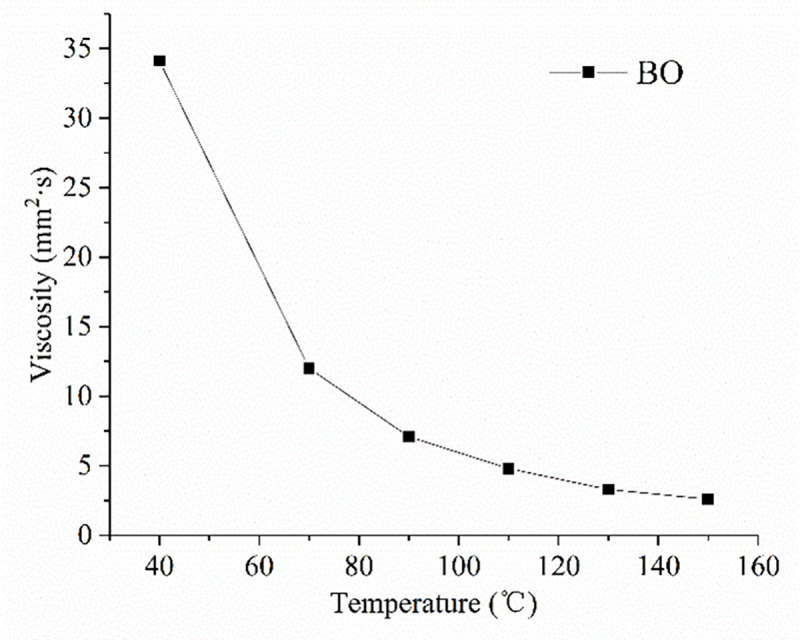
The base oil viscosity in different test temperature.

Prior to experiment, the test specimens were cleaned with petroleum ether and alcohol to remove any contaminants left on the surface. Then, the ring and block were fixed onto the test rig. 20 ml of lubricant was added into the lubricant bath. The test conditions were set according to Table 3. The additive concentration is low, the viscosity of formulated lubricants varies little compared with base oil. Therefore, the base oil viscosity in different test temperature can be the representative of test lubricants, which was shown in Fig 5.

**Table 3:** The friction test conditions.

Temperature	Load	Speed	Testing duration	repetitions	confidence limits of results
40°C, 70°C, 90°C, 110°C, 130°C and 150°C	60 N (approximately 70MPa)	200 r/min	6 hours	4	7%

After experiments, the test specimens were cleaned with petroleum ether and alcohol. The wear scar and surface morphology of test specimens were measured / observed with ZEISS Axio Observer, Chemical elements of friction surfaces were detected by JEOL JSM-7610F Energy dispersive spectrometer (EDS). Tribo-chemical products were detected by Thermo Fisher Scientific K-Alpha 1063 X-ray photoelectron spectroscope (XPS).

## Results and discussion

### Friction and wear

**Fig 6:**
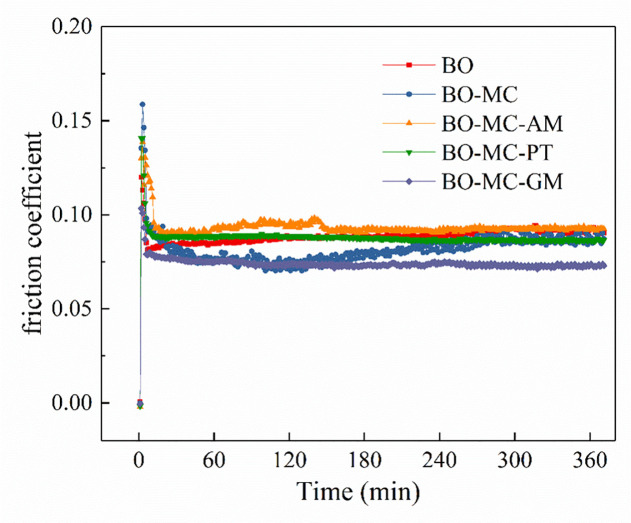
Shows the friction coefficient of test lubricant containing MoDTC and organic friction modifiers during 6 hours test, test temperature 40°C.

Fig 6 shows the friction coefficient variation of lubricants containing MoDTC and organic friction modifiers during 6 hours test, during which the temperature is 40°C. In the initiation, friction coefficient rises sharply to a high level, which is close to 0.16, and then decreases to a relative low value. All the friction coefficients keep stable except for BO-MC, which rises gradually from 0.075 to 0.085 after 280 min test. After 6h test, all the test oil samples show stable value.

**Fig 7:**
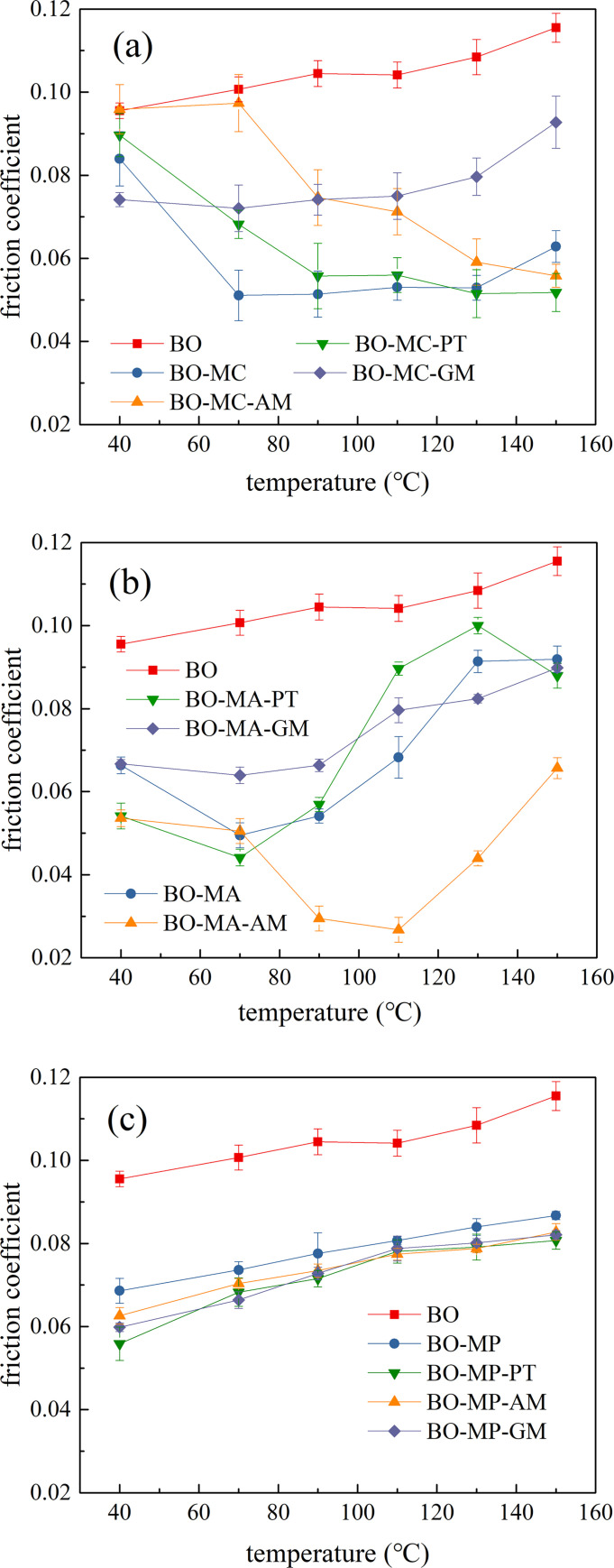
Influence of organic friction modifiers to the friction coefficient of organic molybdenum, (a) MoDTC and organic friction modifiers, (b) MA and organic friction modifiers, (c) MoDDP and organic friction modifiers.

To summarize the friction results and get a clear conclusion, the average friction coefficients of lubricant samples at stable period were illustrated in Fig 7.

Fig 7A shows the influence of organic friction modifiers to MoDTC (MC). In general, with the rise of the test temperature, the friction of BO and BO-MC-GM increases gradually, while the friction of BO-MC-PT, BO-MC-AM and BO-MC drop significantly. Throughout the test, base oil without any additive showed the highest friction coefficient, which rose from 0.094 to 0.116. The BO-MC-GM demonstrated an excellent friction reduction performance at 40°C, before a slight increase from 40°C~130°C. At 150°C, friction coefficient of BO-MC-GM increases from 0.079 to 0.093, which is close to the maximum friction coefficient of BO and BO-MC-AM at 40°C. The friction coefficient of BO-MC declined sharply from 0.084 to 0.051 when temperature rise from 40°C to 70°C, which may be caused by the sparking temperature for the tribo-chemical reaction of organic-molybdenum composition. Then, it increased slightly to 0.062, which may result from the decreasing base oil viscosity with temperature rise. The friction coefficient of BO-MC-PT decreased at a faster pace than BO-MC-AM. Both BO-MC-PT and BO-MC-AM showed a lower friction coefficient than BO-MC at 150°C, which suggests the synergistic effect produced at high test temperatures.

Fig 7B shows the influence of organic friction modifier to Molybdenum amide (MA). The BO-MA, BO-MA-PT and BO-MA-GM show low friction coefficient below 90°C, and then increased sharply to about 0.9. The addition of PT and GM slightly increased the friction coefficient compared with BO-MA. The BO-MA-AM mixture showed a different trend. To be specific, it declined significantly from 0.68 to 0.24 when the temperature increased from 40°C to 110°C. After the friction coefficient reached its minimum at 110°C, the friction coefficient increased almost linearly to 0.068 when temperature rise from 110°C to 150°C, indicating that the addition of AM improved the performance of MA in reducing friction.

Fig 7C shows the influence of organic friction modifier to MoDDP (MP). Generally, the friction coefficient increased with the temperature. The addition of organic friction modifier show synergistic effect with MP, which reduced the friction throughout the testing temperature compared with pure MP. At 40°C, the BO-MP-PT shows the lowest friction coefficient, followed by BO-MP-GM, BO-MP-AM and BO-MP. When temperature was higher than 70°C, the friction coefficient of BO-MP-GM, BO-MP-AM and BO-MP-PT are very close.

**Fig 8:**
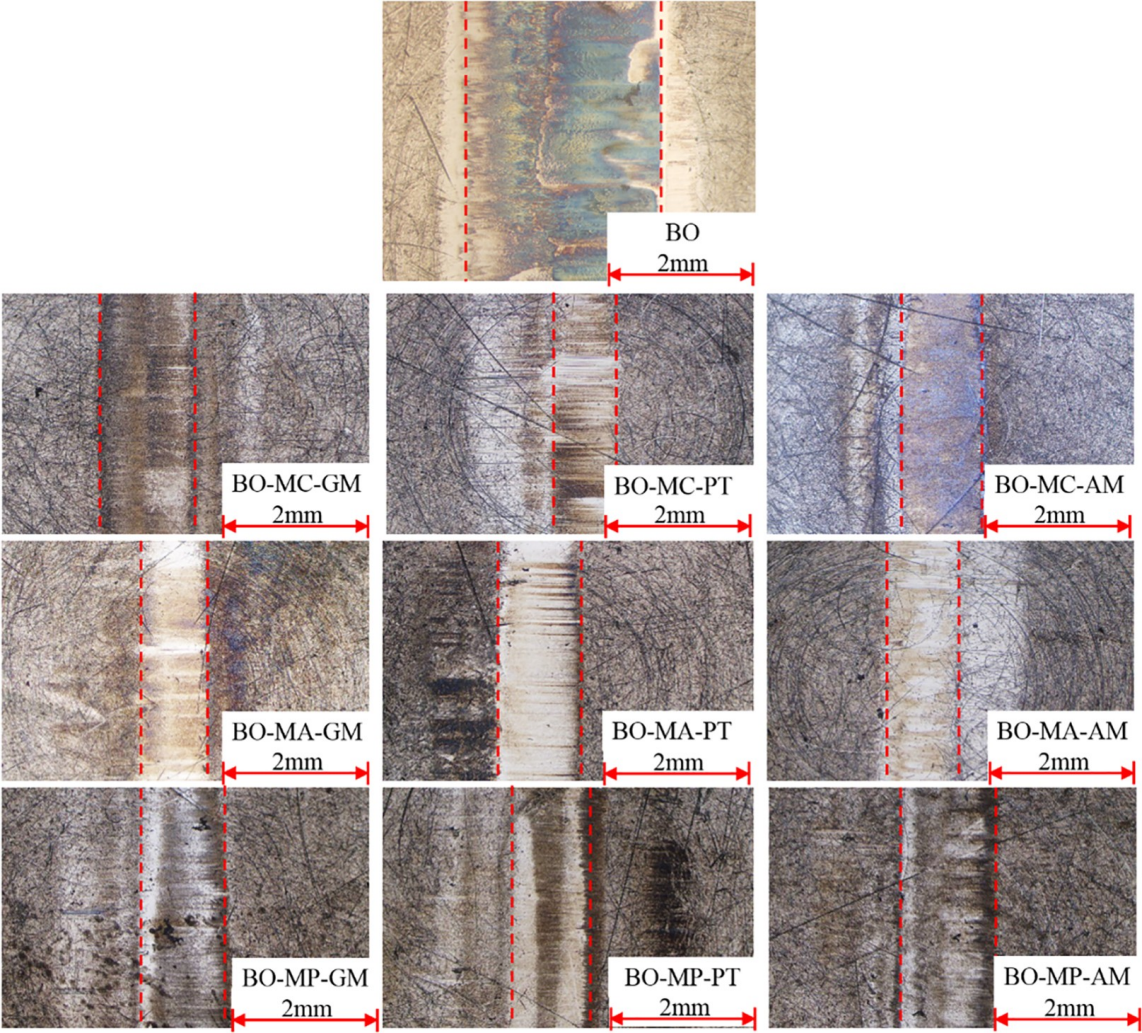
Optical images of block wear surface.

Fig 8 shows the optical images of block wear surface. Because the wear weight measured by analytical balance is not significant during the test, the wear scar width was used to assess the anti-wear performance of different lubricants, which was obtained by measuring the distance between the dash lines. The friction surface of BO show very wide wear scar. The friction surface of BO-MC-AM shows clean friction surface with dark blue color. The surfaces lubricated with BO-MA-GM, BO-MA-PT and BO-MA-AM show clean surface with slight brown wear products. The surface lubricated with other lubricants show dark brown color.

**Fig 9:**
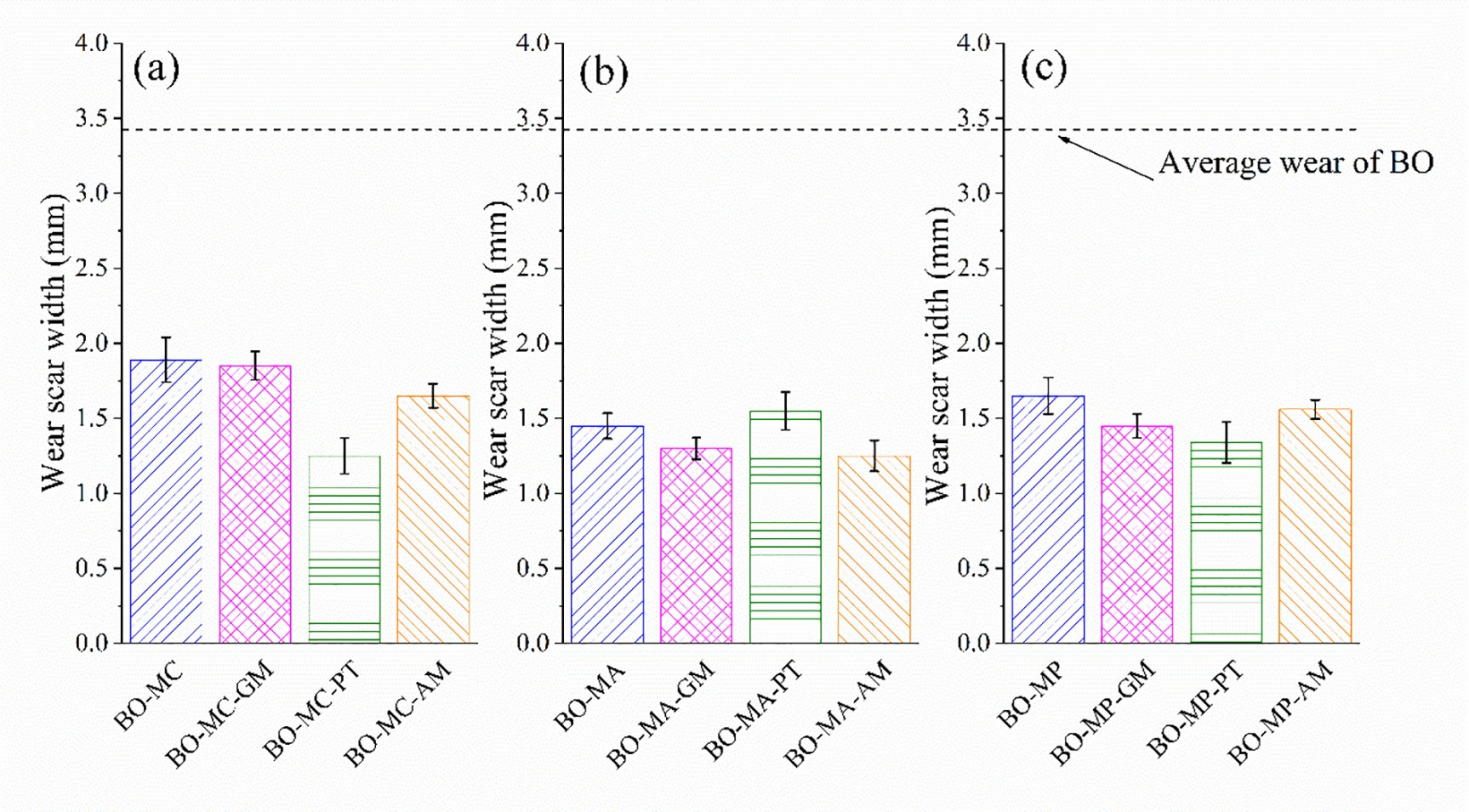
Wear scar width of block lubricated with different test oil formulas at 150°C.

Fig 9 shows the wear scar width of block lubricated with different test oil formulas at 150°C. All the formulations show reduced wear compared with BO. The present of PT reduced the wear of MoDTC and MoDDP obviously. However, the MA containing lubricants show different wear. The present of PT increased the wear of MA.

**Fig 10:**
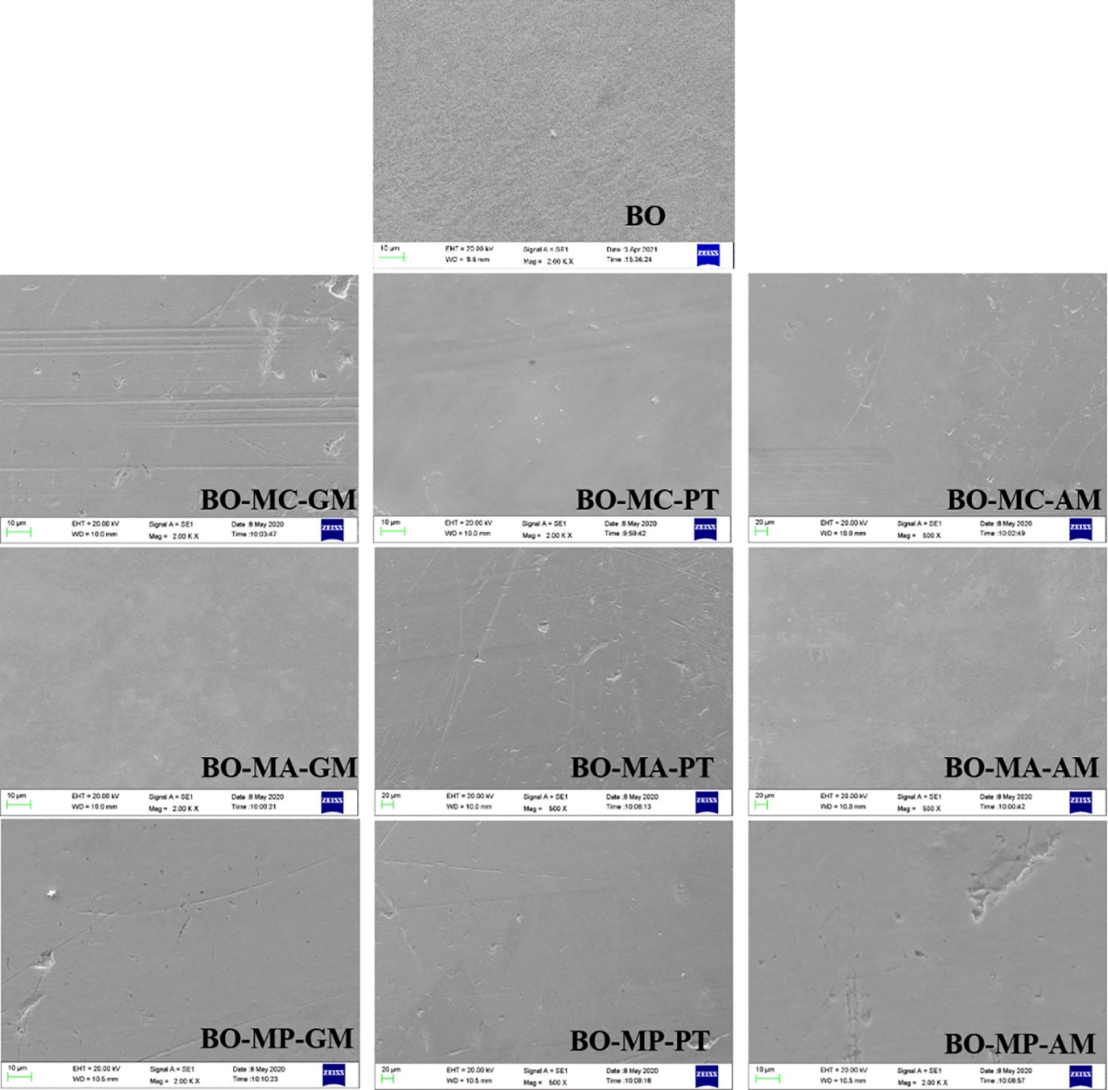
SEM image of wear surface.

Fig 10 shows the SEM image of wear surface. The surface lubricated by BO shows slight adhesive wear with clear surface deformation. However, the BO-MC-GM shows slight scratch, which indicates obvious wear and more severe friction. Other surfaces show flat wear morphology. Some pits can be found on images, which is surface defections. These comply well with the friction tendency in Fig 7 and wear scar width in Fig 9.

## Discussion

**Fig 11:**
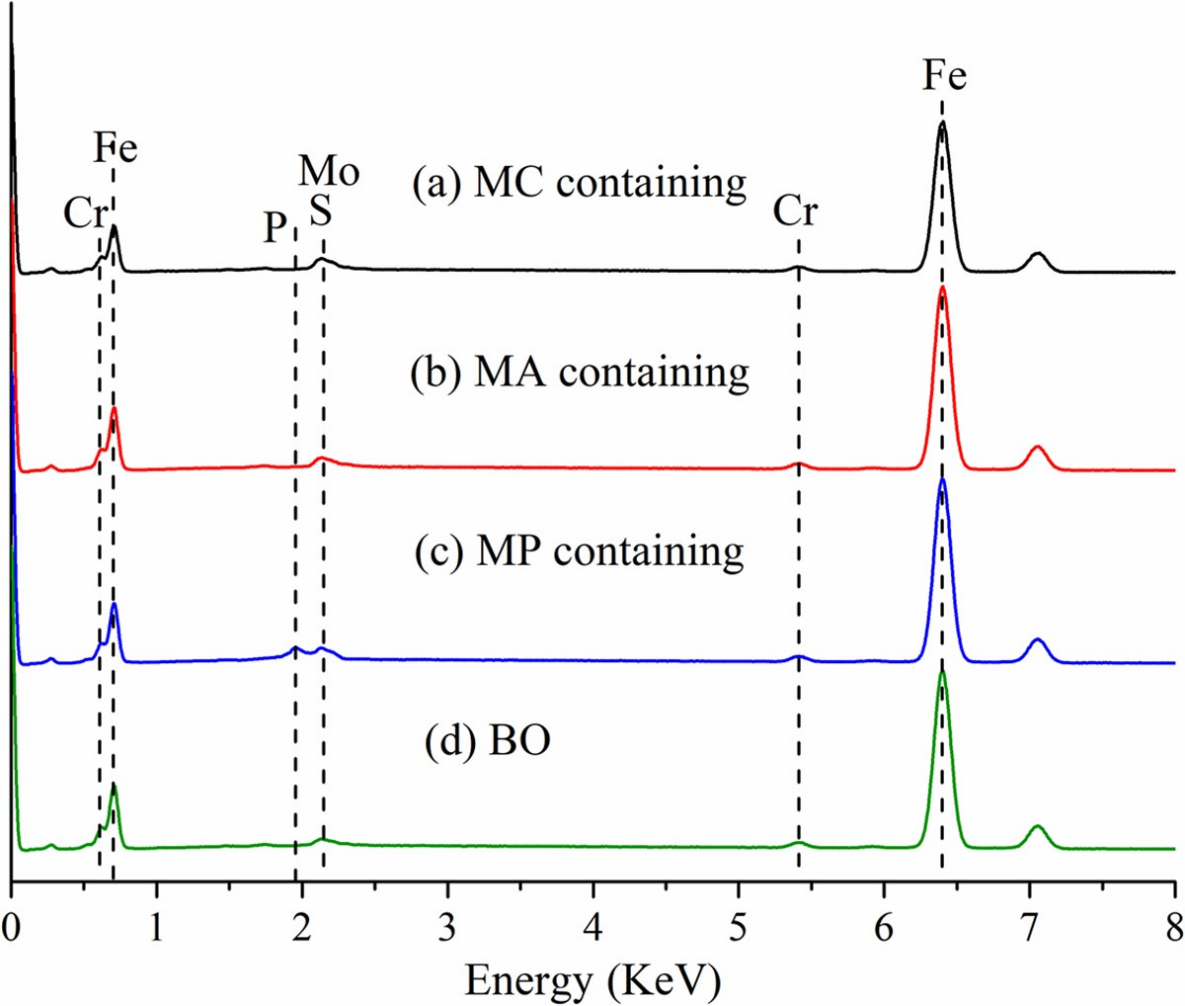
EDS of friction surfaces (a) MoDTC (MC) containing lubricants, (b) molybdenum amide (MA) containing lubricants, (c) MoDDP (MP) containing lubricants, (d) Base oil (BO).

The analysis of EDS and XPS was conducted on wear surface. Fig 11 shows the EDS peaks of the friction surfaces after tests. The friction surface lubricated with MC (Fig 11A) and MA (Fig 11B) mainly contains Fe, Chrome, Molybdenum and Sulfur. The surface lubricated with MP (Fig 11C) contains Phosphorus as well.

The tribo-chemical products were analyzed by XPS photopeaks, and corresponding data were verified with XPS data base [24].

**Fig 12:**
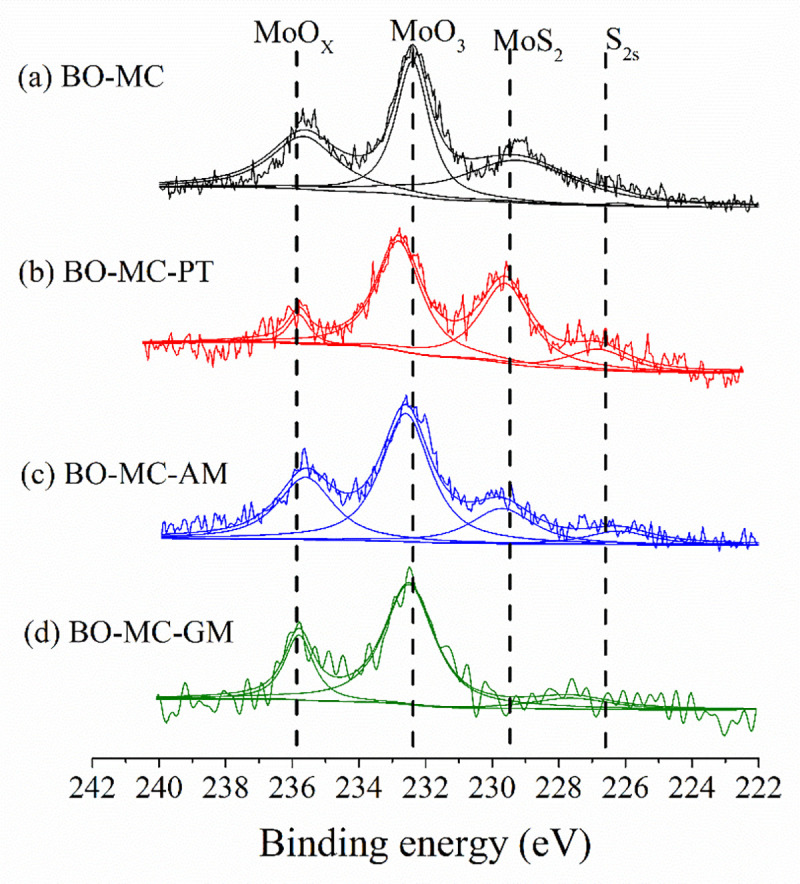
Friction surface XPS photopeaks of Mo3d lubricated with MoDTC (MC) containing lubricants.

The photopeaks of the D orbital in the third electronic shell (Mo3d) were generally used to analyze the chemical composition of Mo containing substance. Fig 12 illustrates the XPS photopeaks of Mo3d lubricated with MoDTC (MC) containing lubricants. Obvious change can be found on the XPS photopeak of Mo3d. In Fig 12A, the wear product of BO-MC mainly composed of MoO_3_ and MoS_2_. In Fig 12B, the MoS_2_ peak in BO-MC-PT is higher than that of BO-MC, which means more MoS_2_ was produced on friction surface in the present of PT. In Fig 12C, the wear product of BO-MC-AM also shows clear MoS_2_ peak, while in Fig 12D, there is nearly no MoS_2_ peak shown for BO-MC-GT. It has been proved that the preferential conversion of organic molybdate to from MoS_2_ can improve the tribological performance [25]. Therefore, the absence of MoS_2_ is the main reason that impact the friction reduction performance of BO-MC-GM. The clear peak of MoS_2_ in BO-MC-PT results in the lowest friction and wear of BO-MC-PT mixture. BO-MC, BO-MC-PT and BO-MC-AM show very low friction coefficient in 150°C, the BO-MC and BO-MC-PT show excellent friction reduction performance throughout the test temperature. Although the anti-wear performance of BO-MC-AM and BO-MC-PT are very low among the test oil formulas, the friction coefficient of BO-MC-AM is relative high until the temperature reach 150°C. Therefore, the best oil formula is BO-GM-PT.

**Fig 13:**
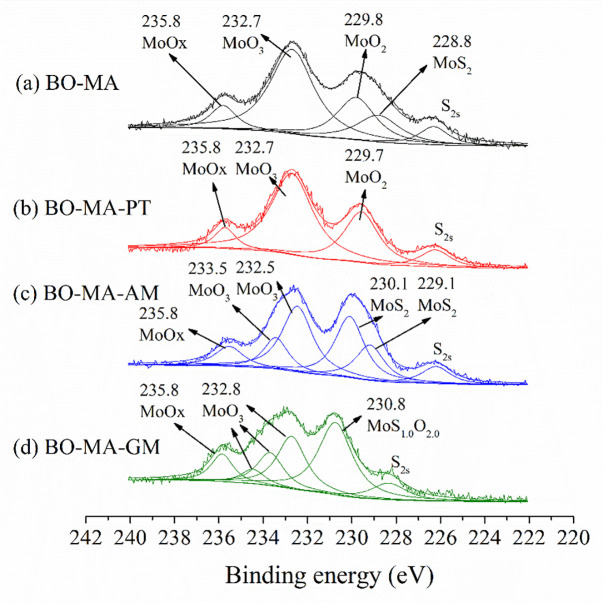
Friction surface XPS photopeaks of Mo3d lubricated with Molybdenum Amide (MA) containing lubricants.

Fig 13 illustrates the XPS photopeaks of Mo3d lubricated with molybdenum amide (MA) containing lubricants. In Fig 13A, the surface lubricated with BO-MA containing MoO_X_ (235.8 eV), MoO_3_ (232.7 eV), MoO_2_ (229.8 eV) and MoS_2_ (228.8 eV). The sulfur present in the friction products mainly comes from the mineral base oil. In Fig 13B, the BO-MA-PT lubricated surface shows MoO_X_ (235.8 eV), MoO_3_ (232.7 eV) and MoO_2_ (229.7 eV). In Fig 13C, the tribo-chemical products of BO-MA-AM containing lubricants containing MoO_X_ (235.8 eV), MoO_3_ (233.5 eV and 232.5 eV), MoS_2_ (230.1 eV and 229.1 eV), the peak in 229.1 eV may also indicate the MoO_2_. In Fig 13D, the BO-MA-GM lubricated surface containing MoO_X_ (235.8 eV), MoO_3_ (232.8 eV) and MoO_1.0_S_2.0_ (230.8 eV).

In conclusion, the results of Fig 13 indicates that the existence of double peak around 230.1 eV and 229.1 eV, indicating MoS_2_, contributes to the low friction coefficient of BO-MA-AM. Although MA contain no sulfur, the contribution of sulfur in the mineral base oil improves the tribological performance of MA. The absence of MoS_2_ leads to the high friction coefficient of BO-MA-PT and BO-MA-GM. The MoS_2_ peak of BO-MA is lower than BO-MA-AM. Therefore, the friction coefficient of BO-MA is lower than BO-MA-AM.

**Fig 14:**
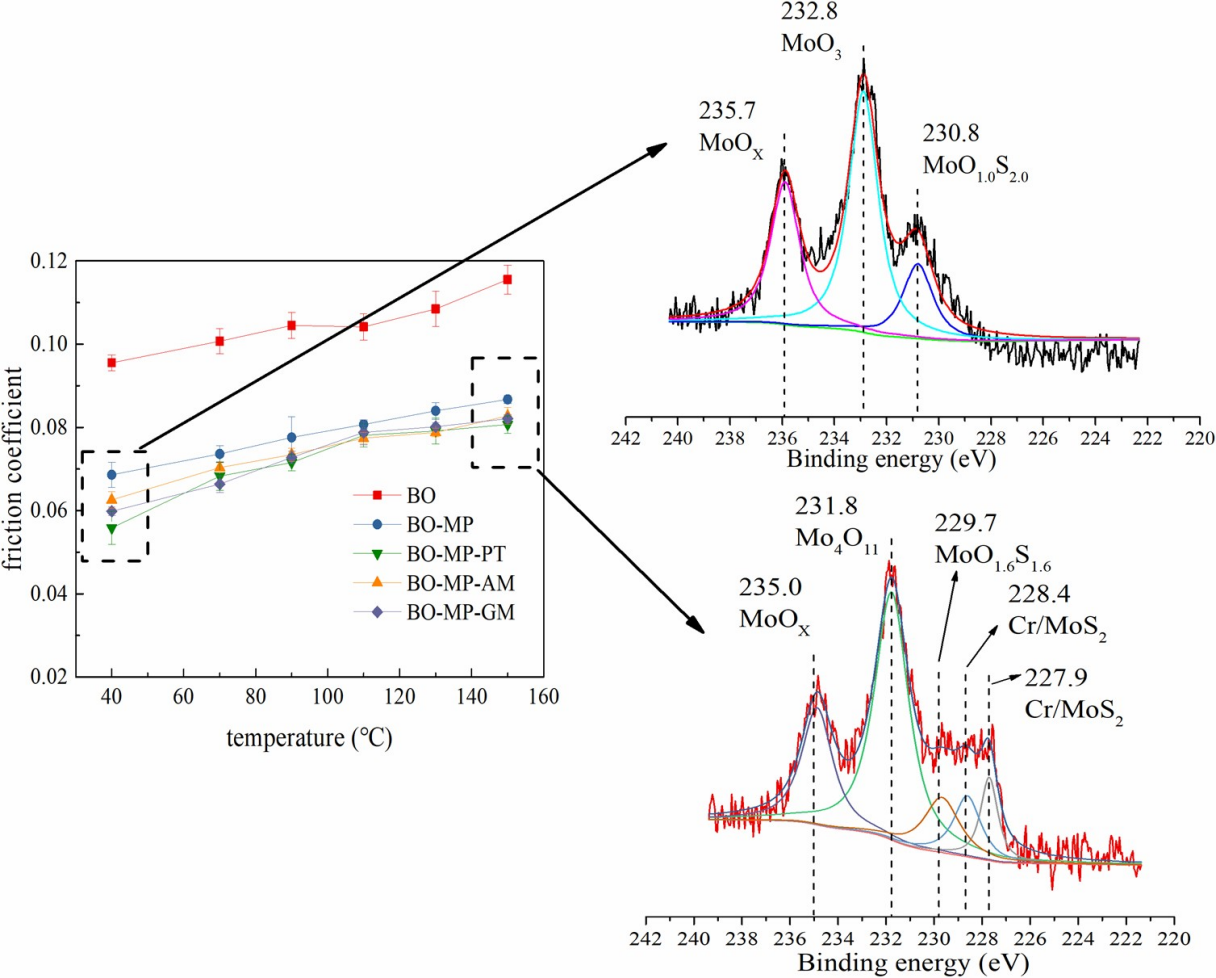
The XPS photopeaks of Mo3d lubricated with MoDDP (MP) containing lubricants.

Fig 14 illustrates the XPS photopeaks of Mo3d lubricated with MoDDP (MP) containing lubricants. In low friction temperature, the friction products of MoDDP containing lubricants are MoO_X_, MoO_3_, and MoO_1.0_S_2.0_. While in high friction temperature, a very wide peak between 230 eV-228 eV can be observed. The main products are MoO_X_ (235.0 eV), Mo_4_O_11_ (231.8 eV), MoO_1.6_S_1.6_ (229.7 eV), Cr/MoS_2_ (228.4 eV, 227.9 eV). The side products of MoO_1.6_S_1.6_, and Cr/MoS_2_ lead to a higher friction coefficient.

## Conclusion

In this paper, an investigation was conducted into the impact of organic friction modifier on the tribological performance of organic molybdenum friction modifier using a block-on-ring test rig, based on which the friction and wear of different additive mixture were recorded and analyzed. The findings of this study are summarized as follows:

1. With the increase of test temperature, the present of GM in MoDTC increased the friction coefficient, while the friction of MoDTC with AM and PT reduced dramatically. The MC-PT is suitable to reduce friction in a wide range of temperature, while BO-MC-AM only shows the lowest friction coefficient at 150°C. BO-MA-AM mixture shows obvious synergistic friction reduction performance. All the tested organic friction modifiers reduced the friction coefficient of MoDDP. All the tested organic friction modifiers reduced the wear of organic molybdenum except for the BO-MA-PT formulation. The PT shows the best anti-wear performance with MoDTC.
2. Synergistic lubrication effect can be found in BO-MC-PT, which shows lower friction coefficient compared with BO-MC-AM and BO-MC. PT promotes the production of MoS_2_ in MoDTC. *N*,*N*-Dimethylhexadecylamine promotes the production of MoS_2_ in molybdenum amide in the present of sulfur containing base oil. In low friction temperature, the tribo-chemical products of MoDDP containing lubricants are MoO_X_, MoO_3_, and MoO_1.0_S_2.0_. In high temperature, the side products of MoO_1.6_S_1.6_, Cr/MoS_2_ and molybdenum hydrocarbon of MoDDP lead to high friction coefficient.
